# Airborne Spread of Methicillin Resistant *Staphylococcus aureus* From a Swine Farm

**DOI:** 10.3389/fvets.2021.644729

**Published:** 2021-06-04

**Authors:** Øystein Angen, Martin Weiss Nielsen, Per Løfstrøm, Anders Rhod Larsen, Niels Bohse Hendriksen

**Affiliations:** ^1^Department of Bacteriology, Parasitology, and Mycology, Statens Serum Institut, Copenhagen, Denmark; ^2^Department of Microbiology and Production, National Food Institute, Technical University of Denmark, Kongens Lyngby, Denmark; ^3^Department of Environmental Science, Aarhus University, Roskilde, Denmark

**Keywords:** MRSA, airborne spread, swine farm, air sampling, zoonotic spread

## Abstract

Spread of livestock-associated methicillin resistant *Staphylococcus aureus* (LA-MRSA) to farmworkers has been recognized as a risk when working in LA-MRSA positive stables, due to LA-MRSA being present on airborne dust particles. Based on this, airborne spread of LA-MRSA through stable vents is a concern that is addressed in this study. The aim of the investigation was to quantify the airborne spread of LA-MRSA from a MRSA positive swine farm. In order to achieve this, a method for sampling large volumes of air was applied. The results were compared to meteorological data and bacteriological investigation of samples from the air inside the swine barn, soil outside the farm, and nasal samples from the individuals participating in the sampling process. MRSA was detected up to 300 m (the maximal measuring distance) from the swine farm in the air but only at low levels at distances above 50 meters (0.085 CFU/m^3^ at a distance of 50 m in the wind plume). MRSA was detected in sock samples obtained at the soil surfaces up to 400 m (the maximal measuring distance) from the farm building. The proportion of MRSA positive soil samples decreased from ~80 to 30% with increasing distance from the farm. A total of 25 human nasal samples were sampled after the farm visits after the participants had stayed in the surroundings of the farm for an average of 10.5 h. When leaving the farm, only two of the samples (8%) were LA-MRSA-positive both obtained from one individual who was the one who had sampled the ventilation shafts. In conclusion, airborne spread of MRSA from swine farms does not seem to be an important route for human contamination for individuals staying a whole working day outside a swine farm.

## Introduction

Farm animals are a reservoir for transmission of livestock-associated methicillin resistant *Staphylococcus aureus* (LA-MRSA) to humans (1–3). The mode of transmission has been of great concern in several European countries, especially those with a low MRSA incidence in humans and large pig productions. In Denmark, the proportion of LA-MRSA positive swine herds has increased dramatically since the first findings in 2008, reaching 95% in 2019 (4) and at the same time a steep increase in human LA-MRSA cases has been observed. This was primarily in persons with direct contact to swine farms; e.g., farm workers and family members. However, approximately one third of LA-MRSA infections in Denmark are not associated with direct livestock contact (5, 6). In 2019, 1,122 new human LA-MRSA cases were registered, including both clinical cases and asymptomatic carriers. Among the new LA-MRSA cases, 34% of the infections and 11% of the colonizations were not associated with direct livestock contact, respectively (4). The transmission to persons without livestock contact most likely occur by human contacts, but may alternatively result from environmental spread of LA-MRSA from swine farms.

LA-MRSA in Danish pigs almost exclusively belongs to clonal complex (CC) 398, predominated by *spa* type t011 and t034, are negative for Panton-Valentine leucocidin (PVL) and do not carry the *scn* gene as marker for the phage encoded human immune evasion cluster (IEC). Staphylococci have been shown to be associated with dust particles in pig farms (7) and LA-MRSA can be cultured from dust particles more than 30 days after sampling (8). Transmission of LA-MRSA to human volunteers with a short-term stay in a contaminated swine farm, has been shown to depend on the concentration of LA-MRSA in air, and only to give a transient contamination in 95% of the volunteers (9). The same study reported airborne MRSA levels from one swine farm in rooms with weaner pigs between 24 and 5.452 CFU/m^3^ and another study on five swine farms presented air concentrations of LA-MRSA between 21 and 517 CFU/m^3^ (10). A German study has shown that LA-MRSA can be detected in low concentrations in air samples close to the farm and in soil samples collected up to 300 meters from swine farms (11). Only limited information is available on the degree of LA-MRSA airborne emissions from swine farms and on the putative risk for transmission to humans staying in farm environments, which has raised concern in some communities neighboring pig farms.

The aim of the current study is to quantify the airborne spread of MRSA from a LA-MRSA positive swine farm. In order to achieve this, a method was applied for sampling large volumes of air by a commercial vacuum cleaner, functioning as an impinger (12). The results were compared to meteorological data and bacteriological investigation of samples from soil and humans participating in the sampling process.

## Materials and Methods

### Description of Farm

The study was performed on an LA-MRSA positive swine farm housing around 2,500 weaned piglets. The building (Supplementary Figure 1) contains 11 sections, eight of these housed weaners aged 4–12 weeks. Each section housed piglets born in the same week, which had been transferred from a sow site located 2 km away. Three sections only contained finishers. In each weaner section there were 10 pens each housing 30–40 piglets. Each section had two ventilation shafts except two of the weaner rooms that only had one ventilation shaft.

The nearest swine farm was located 860m southeast from the research farm.

### Description of Sampling Method

The farm was visited five times from October to December 2018. On two of the visits, the sampling procedure was performed twice (morning and evening) resulting in a total of seven samplings included in the investigation. Bioaerosols were collected from the outlets of the ventilation system of the farm and from the area around the farm by Kärchner DS5800 vacuum-cleaners functioning as impingers as described earlier (12, 13). The sampling was performed as described by Jang et al. (13) with 2 L of PBS in the vortex chamber and the vacuum-cleaner running with a flow-rate of 1.5 m^3^/min.

For collecting bioaerosols from the outlets of the farm ventilation system, the inlet of the vacuum cleaner was connected to a carbon-fiber tube (9m long, 4.5 cm diameter) to be able to reach the outlets at the roof of the farm. Four active ventilator outlets were sampled for 15 min.

At each sampling occasion, bioaerosols were collected downwind from the farm in distances of 50, 100, 200, and 300 m from the buildings. The actual wind direction at the farm used for the placement of the impingers at the start of the sampling day was based on the local weather forecast from Danish Meteorological institute and observations at the locality. At each of the distances 50, 100, and 200 m, four vacuum-cleaners were placed perpendicular to the wind direction with a spacing distance of ~27, 33, and 44 m, respectively, whereas two vacuum-cleaners were placed at a distance of 300 m with a spacing of 56 m between them. Additionally, one vacuum-cleaner was placed 50 m upwind (Supplementary Figure 1). The vacuum-cleaners were placed ~1.5 m above ground level on a platform on top of a step ladder, corresponding to the height of human intake of air and ran for 3 h with a flow-rate of 1.5 m^3^/min, whereby each sampling collected particles from 270 m^3^ air.

After each sampling, the PBS content of the vortex chamber was transferred to Bluecap bottles, kept at 4°C, and transported to the laboratory for analysis within 24 h. Between sampling events, the vortex chamber was washed with 70% ethanol for 5 min followed by wash with sterile deionized water.

### Quantification of MRSA in Air Samples Outside the Farm

MRSA in the liquid samples was quantified by filtration of 10, 100, and 1,000 ml through 0.4μm polycarbonate membranes (Microfunnel, Pall Corporation). The filters were transferred to a Brilliance MRSA 2 agar plate (Oxoid). In addition, 0.5ml of the sample was inoculated on each of two MRSA 2 agar plates. The agar plates were incubated at 35°C for 22–24 h and MRSA positive colonies were counted. MRSA was identified as denim blue colonies. One colony from each sample, picked from the agar plate with fewest MRSA-like colonies, was selected for molecular verification. All MRSA subcultures were verified by a PCR assay detecting *mecA, lukF-PV, scn*, CC398-*hsd*, and *spa* followed by *spa* typing (14). LA-MRSA was identified by the presence of *mecA*, CC398-*hsd*, and *spa* amplicons and absence of *lukF-PV*.

Experiments were performed to investigate the stability of MRSA in the PBS buffer used for air sampling. To test the growth of MRSA under field conditions, an MRSA CC398 field strain (isolate 55-109-3106) was added to PBS to a final concentration of 10^2^ CFU/ml. The amount of MRSA was quantified from ten-fold dilutions on MRSA 2 agar plates after 0, 1, 6, and 24 h incubation at 4°C.

### Air Sampling Within Farm

Airborne MRSA within the farm was sampled on gelatin filters using an AirPort MD8 air sampler (Sartorius, Germany) at a flow rate of 50 l/min. The air sampler was held at a height of ~1.5m above the floor. Between 100 and 500 l of air was sampled to each gelatin filter, which was subsequently transferred to a MRSA 2 agar plate to which the filter adhered and dissolved. Air sampling was performed twice in each section. The MRSA 2 agar plates were incubated at 35°C for 22–24 h starting ~3 h after leaving the farm. The number of colonies was counted and the air concentration of MRSA calculated. One presumptive MRSA-colony per agar plate was confirmed as LA-MRSA as described above.

### Test of Human Samples

Three to six individuals participated in the sampling procedures during the farm visits, where they stayed in an area 0–400 meters from the farm for an average of 10.5 h (range 6–13 h). None of them entered the farm or had direct contact to pigs. At all samplings, the same individual climbed onto the roof in order to position the collection tube in the ventilation outlets.

All personnel involved in the samplings had nasal samples taken by rotating an eSwab™ (Copan) five times in the anterior part of each nostril. Nasal samples were taken immediately after arriving to the farm and just before leaving the farm. All samples were kept at 4°C until cultivation next morning. A total of 25 nasal samples were collected after the farm visits. Presence of LA-MRSA were detected by direct cultivation on Brilliance MRSA 2 agar plates or after enrichment, and putative LA-MRSA were confirmed by PCR as previously described (14).

### Sock Samples

Boot sock (Abena, polypropylene, art. no. 210854) samples were collected at distances of 50, 100, 200, 300, and 400 m downwind from the farm and at 50 m upwind. Soil samples were collected by covering one boot with a clean plastic sock followed by a collection sock and walking 25 meters perpendicular to the line of sight to the farm. The sampling sites were determined by the position of the vacuum-cleaners (see Supplementary Figure 1) and at 400m the starting point was determined by the wind direction similar to the other distances. The socks were transferred to individual sterile plastic bags and kept overnight in a cooled storage container.

Sock samples were transferred to Mueller-Hinton broth (Sigma) supplemented with 6.5% NaCl for overnight enrichments at 35°C followed by spread of 10 μl on Brilliance MRSA 2 agar plates. The plates were read after 20–24 h incubation at 35°C and a denim colony from each sample was confirmed as LA-MRSA by PCR as described above.

### Meteorological Data

Wind speed, wind direction, turbulence and temperature were measured with an ultrasonic (10 Hz Metek, USA-1) on a mast 4.14 m above ground. The mast was placed ~35 m south of the farm building. The data from the ultrasonic are only usable for wind directions from 120 to 255 degrees due to disturbance from vegetation and the farm. For other wind directions, meteorological data from the weather-model WRF were applied from a grid point 25 km east of the farm. WRF (Weather Research and Forecasting model) is a numerical weather prediction system developed by National Center for Atmospheric Research, USA (NCAR) and others (https://www.mmm.ucar.edu/weather-research-and-forecasting-model). The meteorological data were used for estimating the width of the wind plume from the farms during the sampling events. The width of the plume is defined as the span of the wind directions during each of the 3 h long experiments and is calculated as the span of three 1-h averages of the wind direction ± 5 degrees.

## Results

### Bacterial Isolates

All MRSA isolates belonged to CC398, carried the *mecA* gene, they were negative for *scn* and PVL and belonged to either of the *spa* types t011 or t034.

### Air Sampling Within Farm

**Figure 1:**
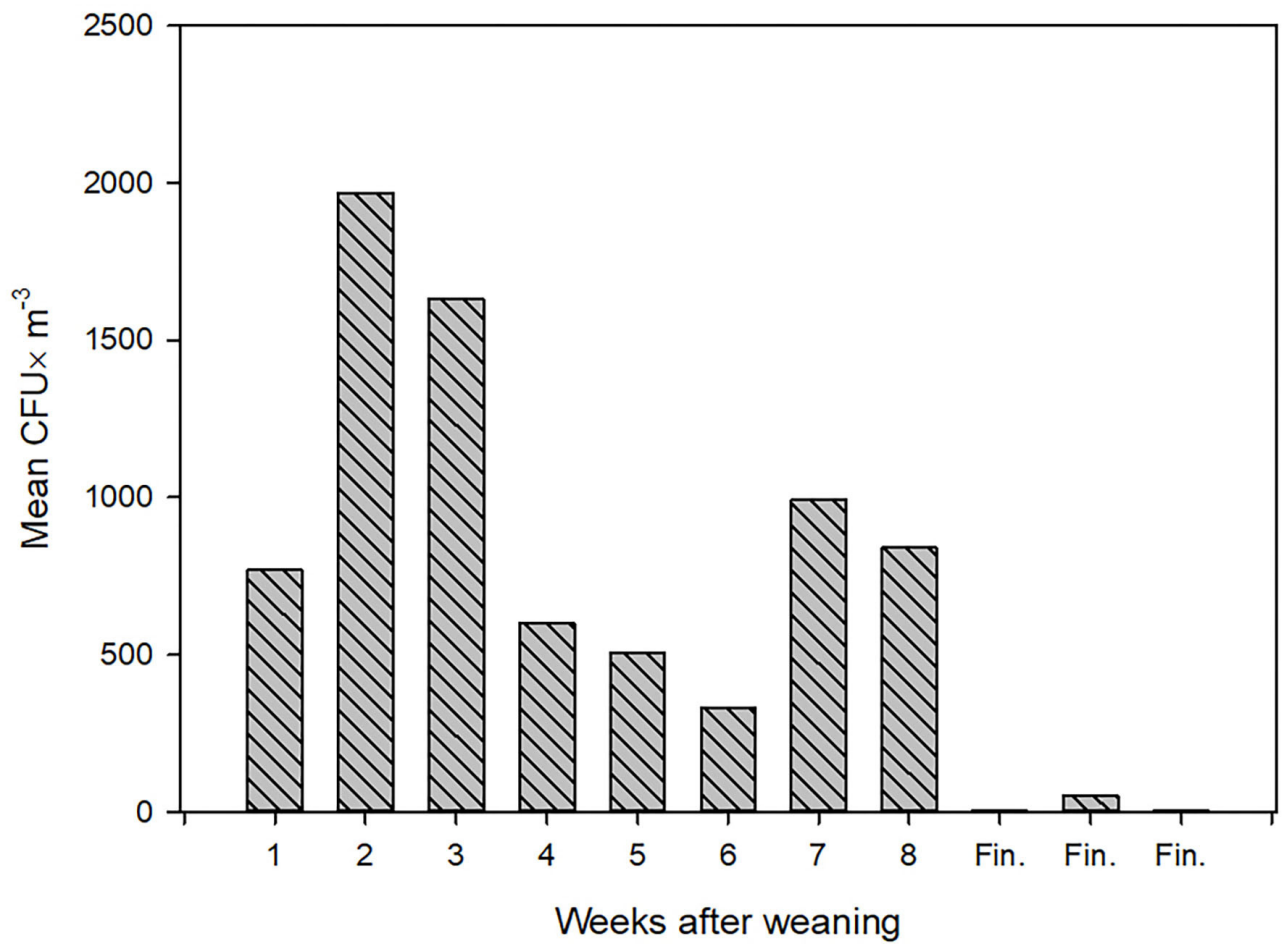
The concentration of airborne LA-MRSA (mean values of two measurements) inside stables among group of piglets of different ages. CFU, Colony Forming Units; Fin., Finishing pigs.

**Figure 2:**
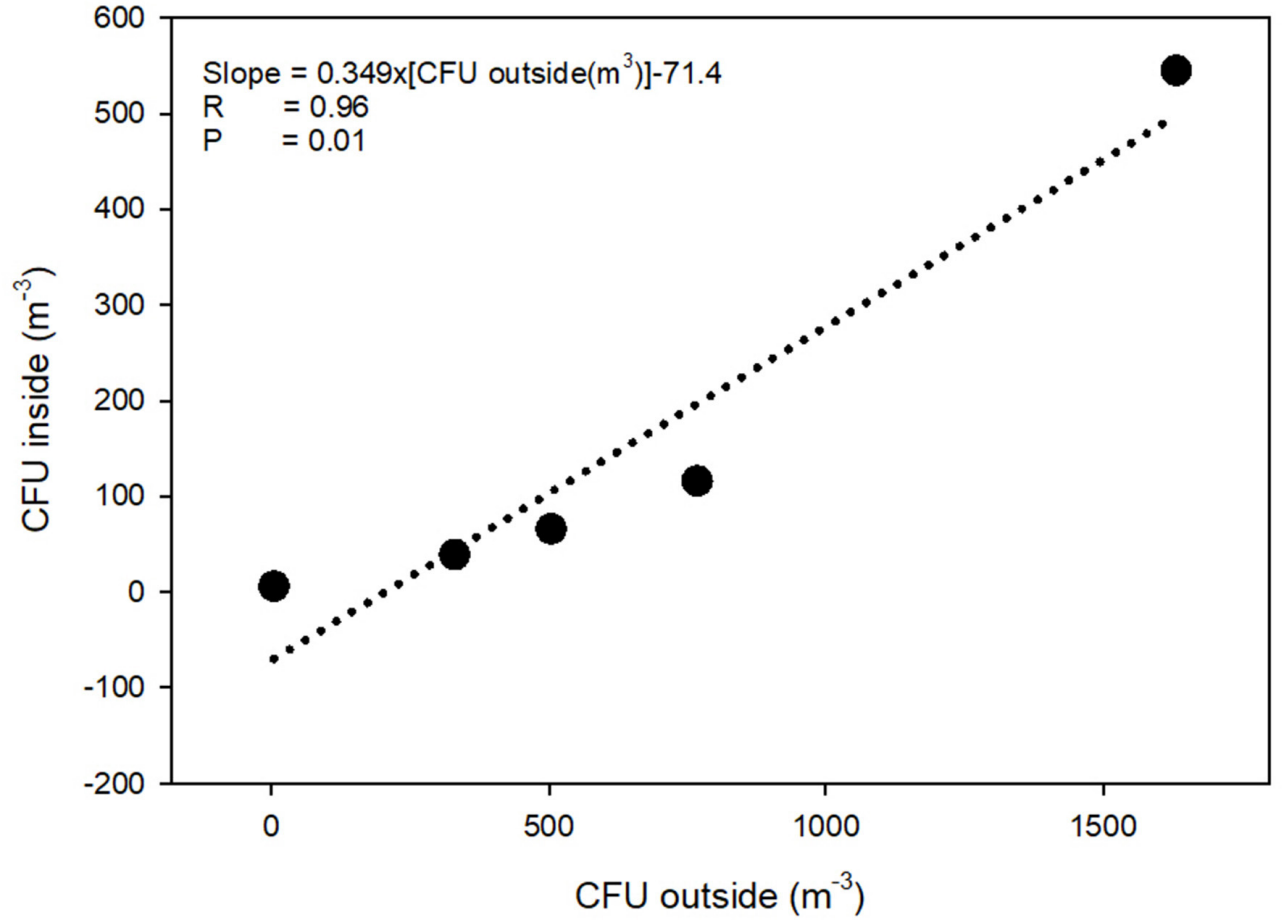
Concentration of airborne LA-MRSA measured inside and from the ventilation shaft outside a swine farm. Data on the two axes are measured by different methods: Gelatine filters (inside) and PBS collection (outside). CFU, Colony Forming Units; R, Pearsson's correlation coefficient.

During one of the visits, the concentration of airborne LA-MRSA was measured twice in the 11 pig sections, eight of these housed piglets 1–8 weeks after weaning and three housed finisher pigs. The amount of airborne LA-MRSA was highest in the units housing piglets 2–3 weeks after weaning and lowest in the finisher sections (Figure 1). Using data from the ventilation system, the amount of LA-MRSA (CFU/h) expelled from the ventilator shafts was computed and compared with the corresponding data obtained from the active ventilator shafts that were sampled from the outside. There was a strong statistically significant correlation (*p* = 0.01; Pearson's correlation) between the values measured inside and in the ventilation shaft outside the farm building (Figure 2).

### Wind Speed, Direction, and Temperature

The wind speed, its direction and the temperature at the five sampling days are listed in Table 1. As expected, at the first and the last sampling occasion the wind speed measured by the ultrasonic was much lower than the WRF-model speed due to the lee effect from the farm. At the three other days, the WRF speeds were a bit higher because data were from 10m height. The wind direction corresponded between the two different datasets and showed variation between 129 and 302°. The wind direction was nearly the same for the first and last sampling occasion, while it differed between the other occasions. The temperature varied from 5 to 16°C over the period of sampling events.

**Table 1:** Wind speed (m/s), wind direction (degrees) and temperature (°C) as calculated from the ultrasonic (4.1 m) and from the weather model (WRF, 10 m).

**Day**	**Wind speed (m/s)**		**Wind direction (degrees)**		**Temperature (^**°**^C)**	
	**Ultrasonic**	**WRF**	**Ultrasonic**	**WRF**	**Ultrasonic**	**WRF**
03-10-2019	4.3	9.0	292	302	12	11
10-10-2019	2.8	3.4	129	136	16	15
06-11-2019	2.3	2.5	173	180	9	7
13-11-2019	5.4	6.6	223	220	10	7
04-12-2019	6.8	11.3	288	293	6	5

*For the first and the last sampling occasion, sonic measurements were disturbed by the farm*.

### MRSA Measurements in Field Air Samples

**Figure 3:**
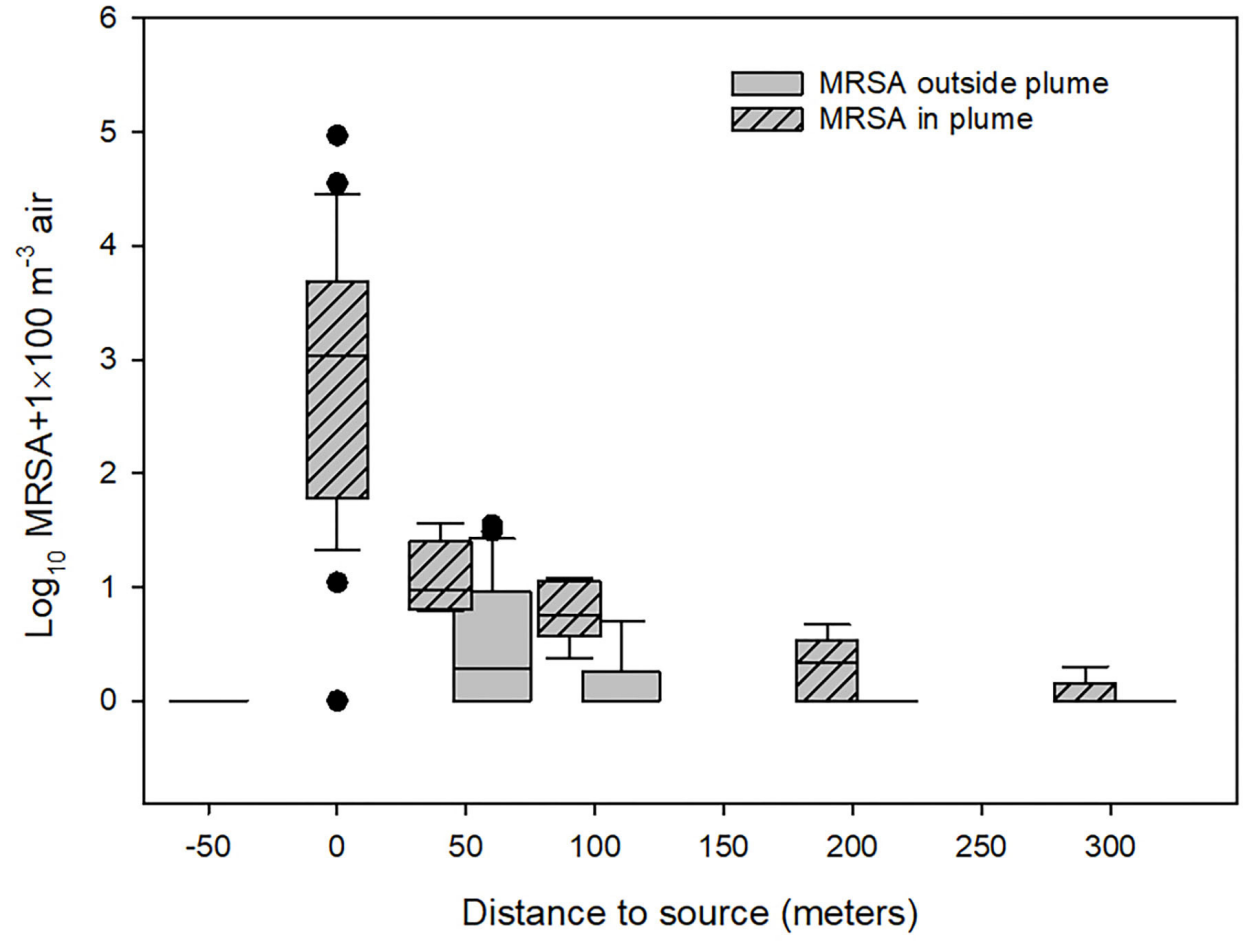
Concentration of LA-MRSA measured outside the swine farm. −50: Measurements 50 meter upwind from ventilation outlets, 0: measurements from ventilation shafts (not including the measurements from empty stables), 50–300: measurements downwind from the farm. The concentration found in the plume at all distances from the farm was significantly higher than the concentration found outside the plume (Mann-Whitney U-tests; *p* < 0.01).

The results of the quantification of LA-MRSA in air samples from the outlets of the farm's ventilation system and in the air around the farm are summarized in the box and whisker-plot shown in Figure 3. It appears from the figure that the concentration of LA-MRSA in the air samples was reduced from 10.9 CFU/m^3^ at the ventilation shaft (median values) to 0.085 CFU/m^3^ at a distance of 50 m in the plume 1.5 m above soil surface, corresponding to a 128-fold reduction, and further to a value close to zero at a distance of 300 m. The variation in the data on the concentration of LA-MRSA found in the outlet from the ventilation system of the farm was significant and varied between 0 (empty stable) and 929 CFU/m^3^. The data was not normally distributed. Further, it appears that LA-MRSA notably exists in the air plume from the farm, as the concentration found in the plume at all distances from the farm was significantly higher than the concentration found outside the plume (Mann-Whitney U-tests; *p* < 0.01). At 50m, the estimated median value was 0.085 CFU MRSA/m^3^ in the plume (min–max: 0.052–0.355) compared to an estimated median value of 0.009 CFU/m^3^ outside the plume (min–max: 0–0.343). The variation was much lower at the larger distances where only few MRSA were detected in the air. The bioaerosols collected 50 m upwind from the farm contained no MRSA. We found no simple relationships between wind speed, wind direction, or the temperature and the aerial dispersal of MRSA. Examples of these observations are shown in Supplementary Figure 2. The wind plumes indicated are averages over the whole observation period and represent wind directions, which might constantly be changing.

In the stability tests it was found that after adding 10^2^ CFU/ml LA-MRSA to a PBS buffer, the CFU count was stable during 24 h at 4°C.

### LA-MRSA in Sock Samples

**Figure 4:**
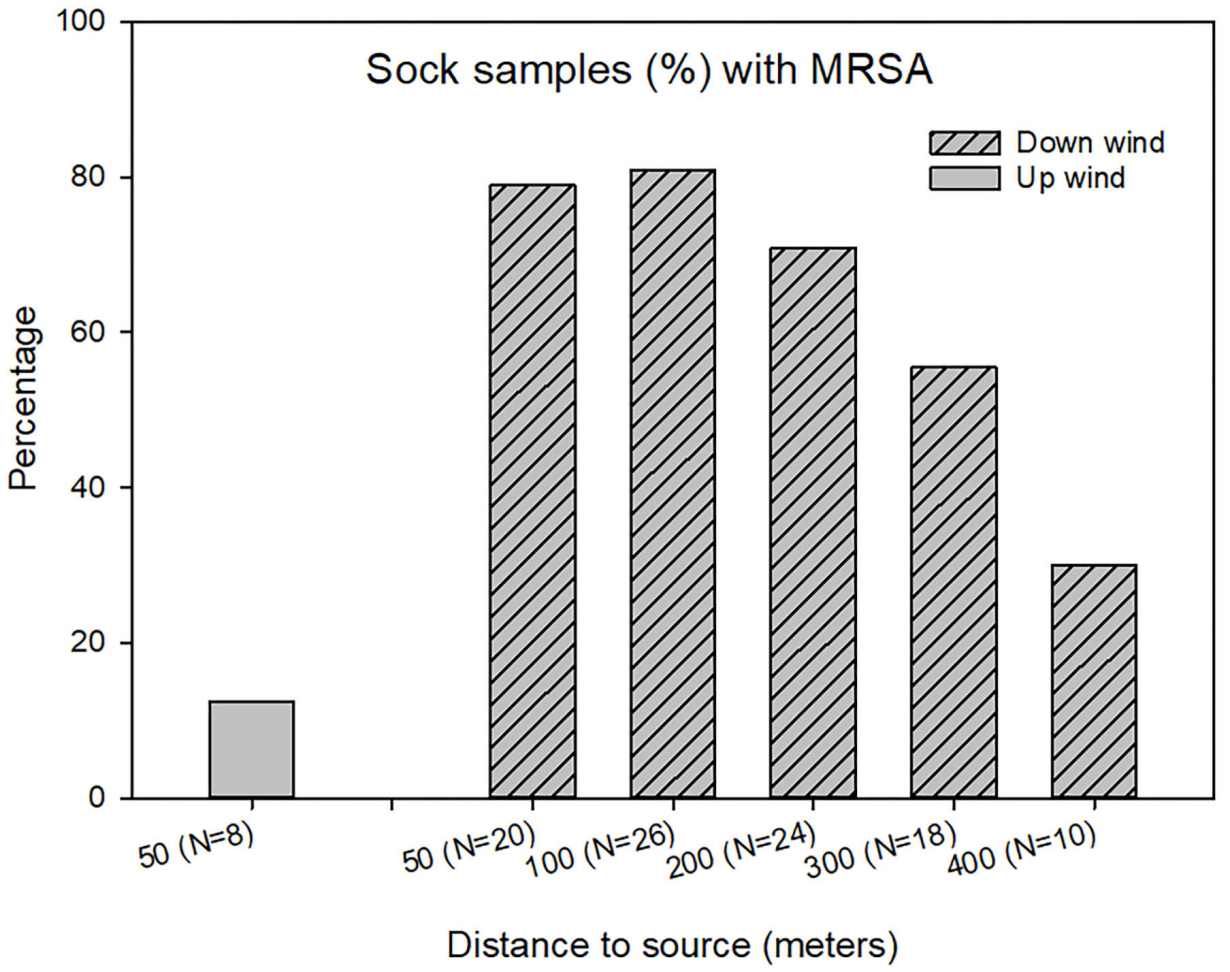
LA-MRSA detected in sock samples outside the swine farm using data from seven samplings. *N* = number of samples at each distance.

LA-MRSA was detected in sock samples obtained at the soil surfaces up to 400m from the farm buildings. The percentage of MRSA positive samples decreased significantly from ~80 to 30% with increasing distance from the farm (Chi-square test, *p* <0.05) as shown in Figure 4. Upwind from the farm, only one of the sock samples (12.5%) was found MRSA positive.

### LA-MRSA in Human Nasal Samples

A total of 25 human nasal samples from 6 individuals were sampled after the farm visits. All participants were MRSA negative when arriving at the farm while two samples (8%) were LA-MRSA positive when leaving the farm. This was in both cases the same person who had been in close contact with the ventilation shafts during sampling.

## Discussion

The investigated farm has been positive for LA-MRSA at least since 2014 (9) and is representative for most Danish pig farms, where 95% of 73 tested farms were positive in 2019 (4). The high number of MRSA positive farms has raised health concerns by people living in proximity to or passing by swine farms. Our study shows that LA-MRSA is indeed present in air collected up to 300m from the farms, but also that the concentration declines steeply by distance from the farm and is very low (0.085 CFU/m^3^ at 50m distance). In accordance with the present investigation, a German study only detected low concentrations (2–14 CFU/m^3^) of MRSA in air samples 50–150 meter downwind from a swine farm (11). The differences in MRSA concentrations compared to the present investigation might be related to differences in farm size, MRSA prevalence, the amount and composition of dust particles, speed of the wind on the days of sampling, and the position of the sampling points relative to the wind plume.

Furthermore, the level of MRSA in swine farms varies considerably, depending on both age of the pigs and their activity, ranging on the same farm between 24 and 5.452 CFU/m^3^ in the weaning units with the highest values among piglets 4–5 weeks after weaning and associated with a high activity level (9). There will probably also be a large variation in shedding level between different farms depending on herd size, ventilation and LA-MRSA level in the pigs.

Our quantification of LA-MRSA exhausted from a swine farm showed a relationship between the MRSA level measured inside the swine farm and in the ventilator shafts, however also that MRSA levels seemed to be three times higher inside the farm than in the ventilator shafts, which may be a result of a dilution caused by the outdoor air as the samples were collected at the top of the shafts.

A number of sampling methods have been applied for quantification of airborne microorganisms, e.g., impaction, impingement, and filtration (15). Filtration sampling is a commonly used method but may result in loss of viability due to desiccation stress during sampling. In this investigation, we tried to minimize the desiccation stress using two methods for indoor sampling (with high MRSA levels) and outdoors (with low MRSA levels), respectively. The high-flow-rate impinger used for outdoor sampling of MRSA has been demonstrated to collect the majority of airborne particles in the size ranges similar to those observed for bacteria. Further, it is concluded by Santl-Temkiv et al. (12) that the Kärcher impinger has advantages for sampling environmental atmospheric bacteria compared to other tested impingers due to its high flow rate, together with its comparatively high retention efficiency and the possibility of long sampling time. In addition, it has been shown that the impinger retained the metabolic activity and viability of the investigated bacteria using sampling periods between 2 and 5 h (12). For short-term sampling within the farm, the bacteria were sampled on to a gelatine filter followed by incubating directly on an MRSA selective agar. This method has earlier been reported to have good efficiency (7, 10, 16). Even though both methods have shown favorable properties regarding bacterial survival, the use of two different methods may influence the comparison of the amount of MRSA in the air inside and outside the farm (as shown in Figure 2).

With increasing distance from the farm, the MRSA level measured in the air declined sharply and was highly dependent on whether the sampling was performed inside or outside the estimated wind plumes (Figure 3). These observations probably mainly represent a dilution effect of the bacteria, as the transport time in the air is probably too short to significantly affect bacterial viability. In some cases the CFU concentration outside the wind plume were relative high, which might be due to incorrect determination of the wind plume, because of uncertainty in the determined wind direction, or complex flow around the buildings. The measurements presented in this paper were all performed during the autumn where bacterial decay might be less effected by UV-light and high temperatures than during summertime, where decay might be expected (17).

A similar dependency on the distance to the farm was observed for the sock samples, where only a few sock samples were found positive 400 meters from the farm. This corresponds to the observation by Schulz et al. (11) where MRSA was detected in low frequency in soil samples up to 300 meters from swine farms. As MRSA are constantly deposited to the farm surroundings every day of the year, these low levels might indicate that MRSA only have limited survival on the soil surface. Presence of MRSA in the soil might also be related to spread of manure containing MRSA from the farm. Manure was spread on the soil in early spring, almost 6 months before the start of the samplings, and was therefore not an obvious source of LA-MRSA in the soil. Previous studies has shown little accumulation of bacteria in farm soil where manure has been spread early (18) so this effect is assumed to be insignificant, whereas other vectors as rodents and flies could influence the spread of LA-MRSA to the farm surroundings.

An attempt to interpret these results into risk assessments to respond to the public concern is a difficult challenge. Even though only low levels of MRSA were observed at a distance of 50 meters from the swine farm one might speculate whether this nevertheless might be of public concern. Transmission of LA-MRSA to human volunteers has earlier been shown to be dependent on the concentration of MRSA in the air (9). However, the nasal LA-MRSA contamination was found to be of transient nature after a short-term (1 h) stay in a LA-MRSA positive swine farm. A tentative conclusion is that human colonization is dependent on repeated exposure to LA-MRSA over extended time periods and will not be observable in short-term investigations. In another Danish study, volunteers were sampled in five different swine herds in order to estimate the airborne dose of MRSA necessary for nasal colonization after staying within the swine unit for 1 h. Also here the colonization was found to be of a transient nature and it was not possible to establish a stable estimate for the colonization dose, mainly due to high variation in the measurements of airborne MRSA (10).

In the present investigation, nasal samples were obtained from the people involved in the sampling activities around the farm. At each sampling event, they stayed for an average of 10.5 h outside the farm building and up to 400 meters away from the farm, most of the time around 50–100 meters from the farm, where the majority of the samples were gathered. Out of the 25 nasal samples collected immediately after the sampling period, only two samples were found LA-MRSA positive, both obtained from one individual who was the one who had sampled the ventilation shafts. This indicates that the degree of LA-MRSA contamination of humans staying in the surroundings of a swine farm is limited and temporary. In support of this, it has been shown that living within a distance of up to 2 km away from a pig farm does not increase the risk of becoming infected by LA-MRSA compared to living at distances up to 5 km away (19).

In conclusion, our study shows low level of MRSA in air samples taken in the surroundings of an LA-MRSA positive swine farm. An interpretation of the data is therefore that only people living in close vicinity (<50m) or at the farm are expected to be exposed to LA-MRSA at quantities that may be of concern. Risk of LA-MRSA spread from soil surfaces to humans, remain unanswered by our study, but seems rather speculative.

## Data Availability Statement

The original contributions presented in the study are included in the article/Supplementary Material, further inquiries can be directed to the corresponding author/s.

## Ethics Statement

Collection of bacteria from nasal samples from human participants have been reviewed by the National Committee on Health Research Ethics (journal no. H-21000591) who concluded that ethical approval could be waived for this project. Informed consent was obtained from all participants.

## Author Contributions

ØA: conceptualization of study, field sampling, analysis of data, and writing initial draft. MN: conceptualization of study, field sampling, and reading and editing manuscript. PL: data analysis and reading and editing manuscript. AL: conceptualization of study and reading and editing manuscript. NH: conceptualization of study, field sampling, and reading and editing manuscript. All authors contributed to the article and approved the submitted version.

## Conflict of Interest

The authors declare that the research was conducted in the absence of any commercial or financial relationships that could be construed as a potential conflict of interest.
