# Supplementary material for: Airborne Spread of Methicillin Resistant Staphylococcus aureus From a Swine Farm
Source: Front Vet Sci. 2021 Jun 4;8:644729. doi: 10.3389/fvets.2021.644729 (PMC8211894; doi:10.3389/fvets.2021.644729)
Supplement: Supplementary file 1 [file Data_Sheet_1.docx]

**Supplementary Figure S1**


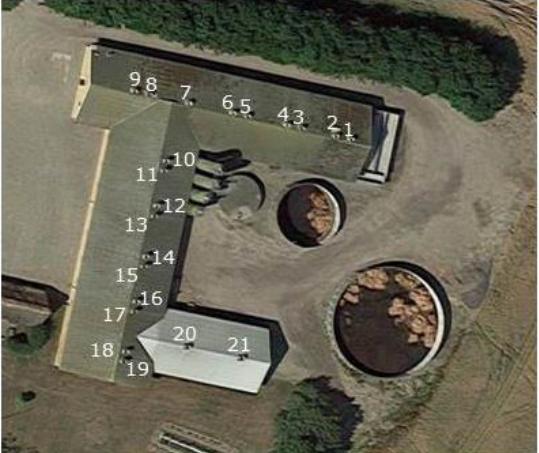


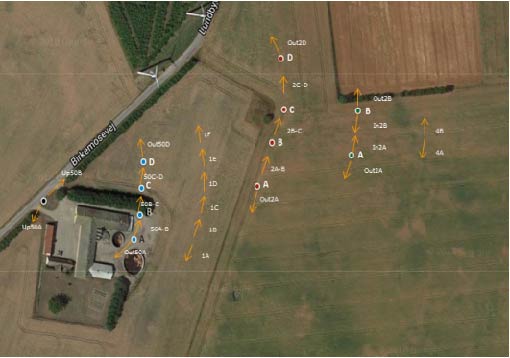


A. Photographic overview of farm with indication of ventilation shafts on the roof (n=21).

B. Example of set up for environmental sampling. Full circles: impingers, arrows: sock sampling. 4A/4B: collection of sock samples at 400 m.

**Supplementary Figure S2**

LA-MRSA air measurements in field air samples, observations from two sampling events are shown. Only sampling event 1 from each day was used in the data analysis. The upper graphs show registration of wind speed measured by ultrasonic and WRF, red lines indicate the time interval of LA-MRSA measurement. The lower graphs indicate the wind plumes during the observation period (span of three one-hour averages of the wind direction ± 5 degrees) and the LA-MRSA measurements (CFU/m^3^). Blue circles: location of samplers, yellow circles: outlet of ventilation shafts, red triangle: meteorological mast.

| October 3. | 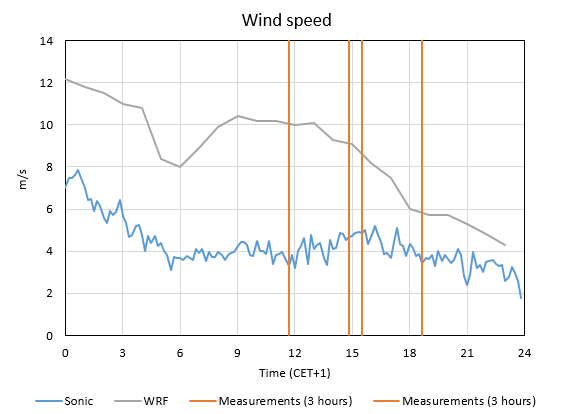 |
| --- | --- |
|  | 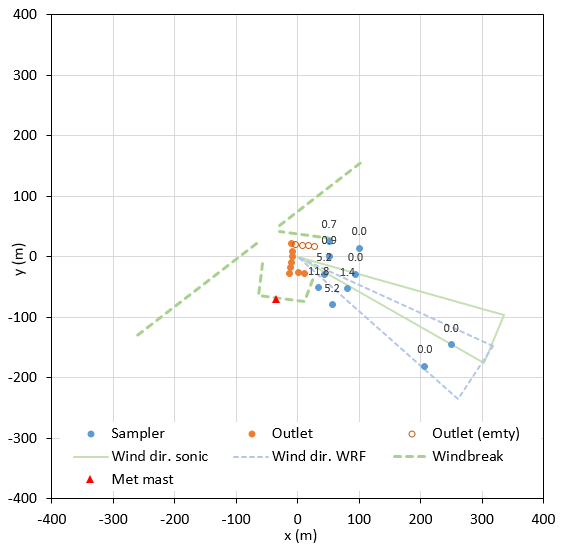 |

| October 10. | 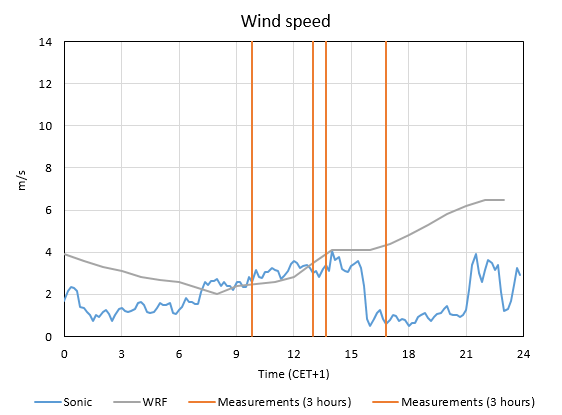 |
| --- | --- |
|  | 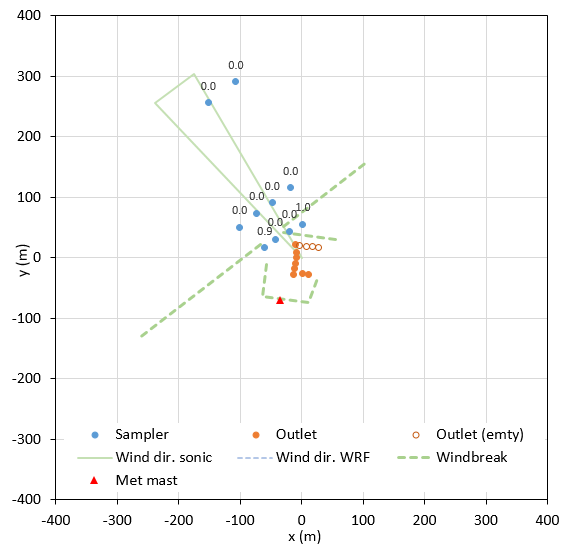 |

| November 6. | 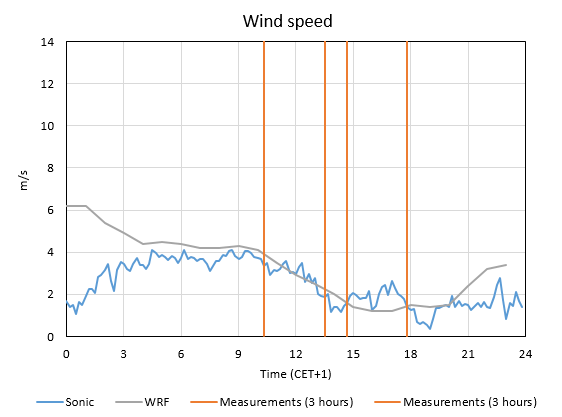 |
| --- | --- |
|  | 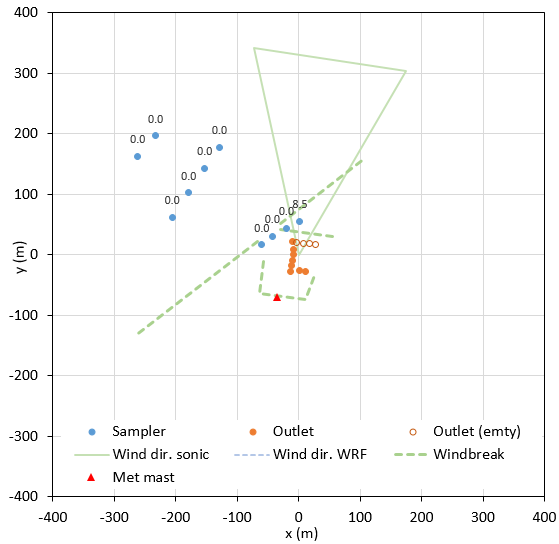 |

| November 13. | 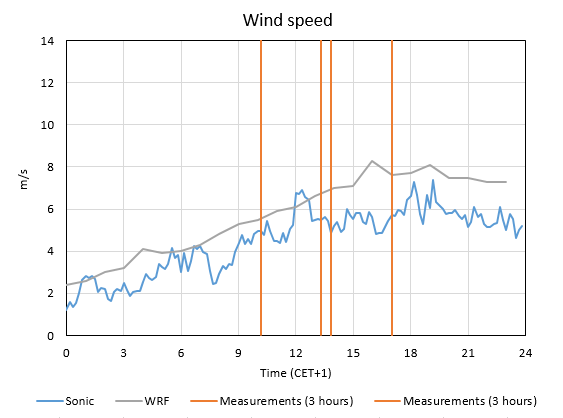 |
| --- | --- |
| 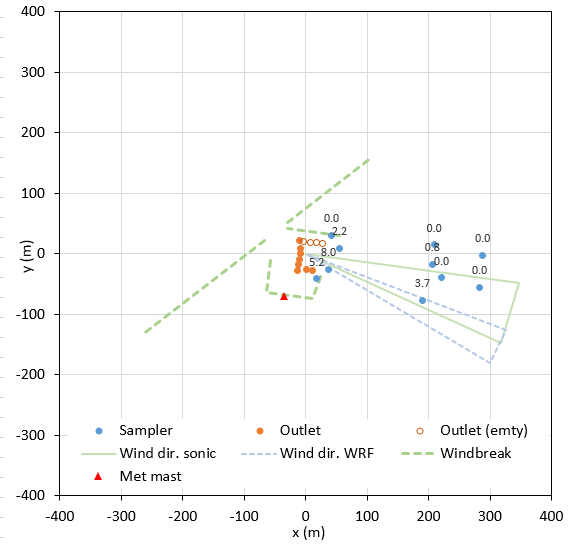Sample 1 | 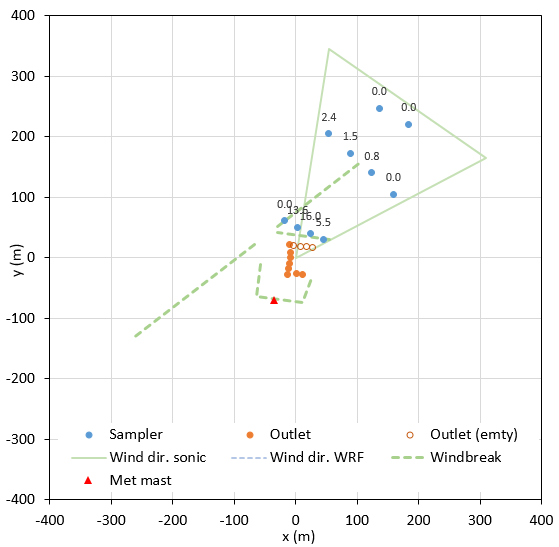Sample 2 |

| December 4. | 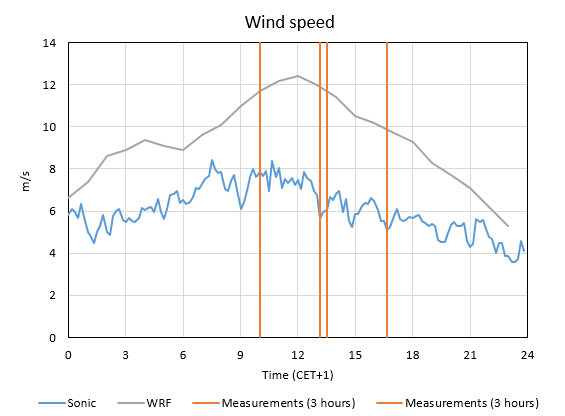 |
| --- | --- |
| 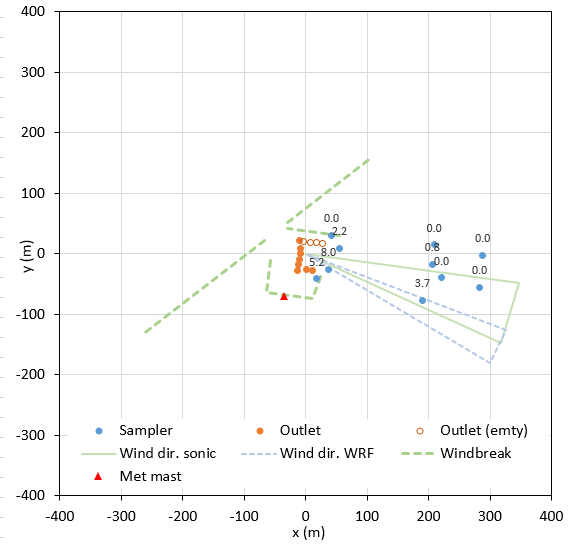Sample 1 | 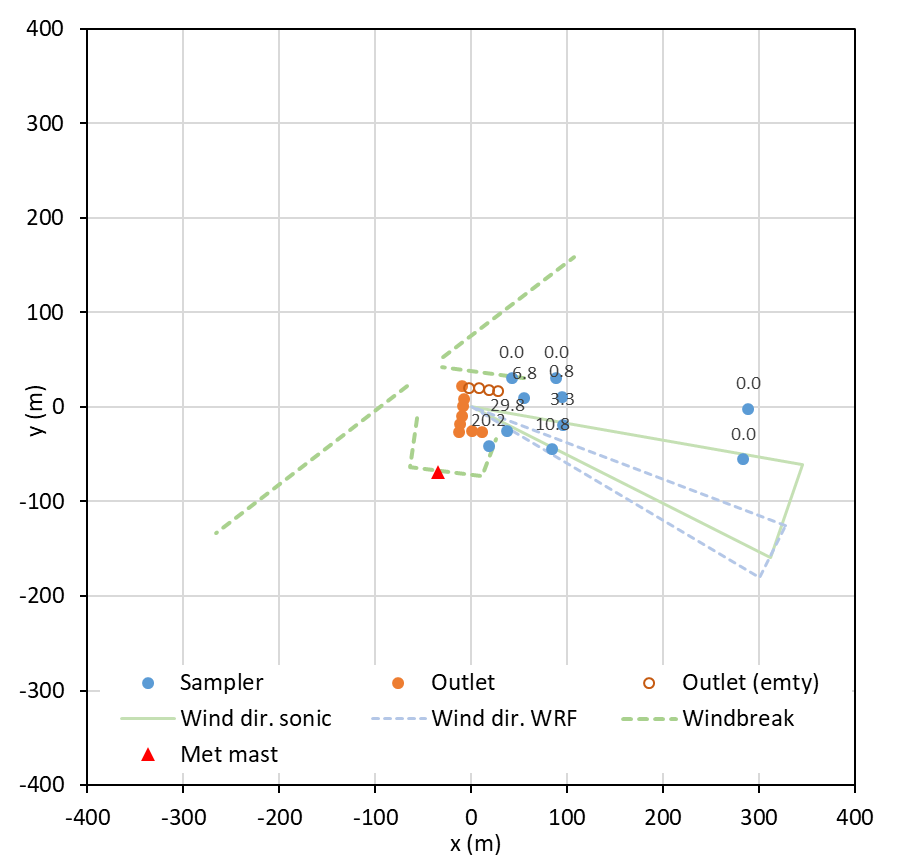Sample 2 |
